# Dietary Patterns and Circadian Syndrome among Adults Attending NHANES 2005–2016

**DOI:** 10.3390/nu15153396

**Published:** 2023-07-31

**Authors:** Zoha Akbar, Zumin Shi

**Affiliations:** Human Nutrition Department, College of Health Sciences, QU Health, Qatar University, Doha P.O. Box 2713, Qatar; za1404491@qu.edu.qa

**Keywords:** diet, dietary patterns, circadian rhythms, circadian syndrome, NHANES

## Abstract

The study aimed to assess the associations of dietary patterns and circadian syndrome (CircS). Data from National Health and Nutrition Examination Survey (NHANES) 2005–2016 were analyzed (*n* = 10,486). Factor analysis was used to construct dietary patterns based on two 24 h food recalls. CircS was defined based on components of the metabolic syndrome, with the addition of short sleep and depression symptoms. Multivariable logistic regression was used to analyze the associations. Two major dietary patterns were identified. The Western dietary pattern had high loadings of refined grains, solid fats, added sugars, and red and cured meats, while the prudent pattern was characterized by a high intake of vegetables, whole grains, oils, nuts, and seeds. The prevalence of CircS was 41.3%. Comparing extreme quartiles of intake, the odds ratios (OR) for having CircS were 1.96 (95%CI 1.53–2.53) and 0.71 (95%CI 0.58–0.86) for the Western pattern and prudent pattern, respectively. The association between the Western dietary pattern and CircS was stronger among men (OR = 2.05; 95%CI 1.48–2.85) and those with low income (OR = 1.94; 95%CI 1.27–2.96) and high education (OR = 3.38; 95%CI 1.90–6.04). The Western dietary pattern was associated with a higher likelihood of having CircS, while the prudent pattern was inversely associated with CircS.

## 1. Introduction

Cardiometabolic risk factors that are grouped together as Metabolic Syndrome (MetS), including central obesity, dyslipidemia, impaired glucose tolerance, and hypertension, continue to rise in prevalence worldwide [1]. Insulin resistance [2], genetics [3], and obesity-driven inflammation [4] have been proposed in the etiology of MetS. However, due to a lack of consensus on the etiology, the concept of Circadian Syndrome (CircS) was introduced, as increasing evidence shows that disruption in circadian rhythms may be responsible for type 2 diabetes mellitus (T2DM), cardiovascular disease (CVD), and hypertension, in addition to commonly co-occurring conditions such as sleep disturbances, depression, and nonalcoholic fatty liver disease (NAFLD) [5,6,7]. CircS expands on the existing criteria for MetS to include depression, sleep disorders, and NAFLD. Studies demonstrate that CircS is a better predictor than MetS for CVD, as well as lower urinary tract symptoms and benign prostate hyperplasia in men [8,9]. CircS also appears to be a mediator between prolonged air pollution exposure and CVD [10]. Furthermore, CircS is associated with increased odds of stroke [11] and testosterone deficiency [12], as well as kidney stones in overweight individuals [13].

Circadian rhythms are produced by the internal circadian clock which consists of a central clock in the brain and peripheral clocks in the organs. These rhythms are generated via transcription factors and gene expression, and regulate key physiological functions in the body, such as metabolism [14,15,16]. The circadian clock also regulates peripheral body clocks present in virtually every organ of the body, such as the liver, heart, muscle, and adipose tissue [17]. These rhythms are influenced by environmental signals including light, exercise, temperature, and food intake. Studies on animal models have shown that high-fat diets disrupt circadian rhythms [18,19,20]. In humans, these findings are corroborated by evidence of impaired glucose tolerance on a high-fat Western-style diet [21] and increased CVD risk with the consumption of animal protein and low-quality carbohydrates in dinner [22,23].

Eating habits play a key role in determining health status and the likelihood of developing chronic illness. An unhealthy diet is linked with increased rates of morbidity and mortality [24], whereas dietary patterns rich in whole grains, fruits, vegetables, unsaturated fats, and lean meats that minimize the intake of processed foods, saturated fat, added sugar, and refined grains reduce the risk of chronic disease [25]. 

Currently, no study has examined the relationships between dietary patterns and the prevalence of CircS. Using data from the National Health and Nutrition Examination Survey (NHANES), we aimed to (1) examine the association of dietary patterns and CircS in US adults, and (2) test whether the association is modified by sociodemographic and lifestyle factors.

## 2. Materials and Methods

### 2.1. Study Design and Sample

The study used data from six cycles of NHANES between 2005 and 2016. NHANES is a nationally representative study that uses a complex, multistage, probability sampling design. The continuous NHANES started in 1999 and is conducted annually via a series of surveys, physical measurements, and medical, dental, and biochemical assessments to evaluate the health and nutritional status of the US population. 

**Figure 1:**
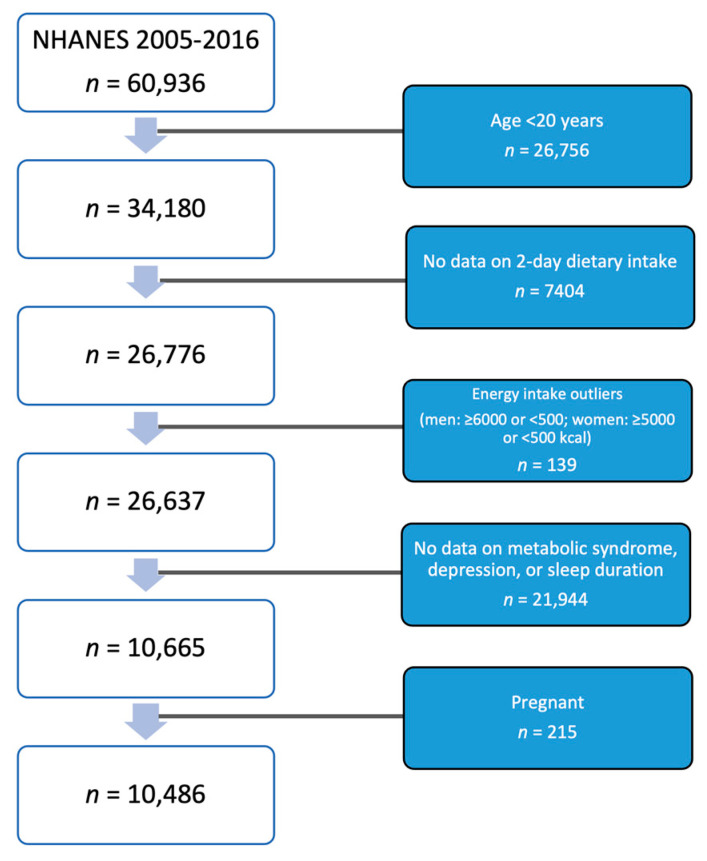
Sample flowchart. Abbreviations: NHANES, National Health and Nutrition Examination Survey.

The combination of the six NHANES cycles between 2005 and 2016 created an analytical sample of 10,486 adults aged 20 and above (Figure 1). The selection of NHANES waves was to complement the previous study [26]. The following exclusion criteria were used to generate the analytical sample: (1) missing data on two-day dietary intake; (2) implausible energy intake (defined as men with an intake of <500 kcal and >6000 kcal, and women with <500 and >5000 kcal intake); (3) subjects with missing values of CircS components; (4) pregnant women, as pregnancy is associated with irregular sleeping patterns and various hormonal and metabolic changes [27,28].

### 2.2. Outcome Variable: Circadian Syndrome

Anthropometric and biochemical measurements were taken by trained examiners in the mobile examination centers (MEC) and during home examinations. The assessment of CircS was based on the criteria by Shi and colleagues [8] which includes the components of MetS, short sleep, and depression. MetS was defined using the criteria set by the International Diabetes Federation Task Force on Epidemiology and Prevention, National Heart, Lung, and Blood Institute, American Heart Association, World Heart Federation, International Atherosclerosis Society, and International Association for the Study of Obesity [29]. CircS was defined as the presence of 4 or more components out of 7 based on the literature [8]: elevated waist circumference, elevated fasting glucose (or on medication), elevated blood pressure (or on antihypertensive medication), reduced high-density lipoprotein cholesterol (HDL-C) (or on lipid medication), elevated triglycerides (or on lipid medication), short sleep, and depression symptoms. 

Components of CircS were defined as follows:

1. Elevated waist circumference was defined by waist circumference ≥88 cm for women and ≥102 cm for men.
2. Elevated fasting glucose was defined by fasting glucose ≥100 mg/dL, or patients being treated with medication, which was assessed with the question “now taking diabetic pills to lower your blood sugar?”
3. Elevated triglyceride was defined by serum triglycerides ≥150 mg/dL, or patients being treated with medication, which was assessed with the question “because of your high blood cholesterol, have you ever been told by a doctor or other health professional to take prescribed medicine?”
4. Reduced HDL-Cholesterol was defined by serum HDL-C <40 mg/dL in men and <50 mg/dL in women or, the answer “yes” to the question “to lower blood cholesterol, ever been told by a doctor or other health professional to take prescribed medicine?”
5. Elevated blood pressure was defined by systolic blood pressure (SBP) ≥130 mmHg or a diastolic blood pressure (DBP) ≥85 mmHg, or patients being treated with antihypertensive medication. The use of antihypertensive medication was assessed by the survey question “Are you now taking prescribed medicine for HBP?”
6. Short sleep was defined by a sleep duration of <6 h per day [30] and was measured by the question “How much sleep do you usually get at night on weekdays or workdays?”
7. Depression symptoms were based on a score of ≥5 of the Patient Health Questionnaire (PHQ-9). It is a 9-item depression screening instrument that aims to evaluate the occurrence of depression symptoms within the preceding two weeks. The scores are categorized as no/minimal depression (score of 0–4) or depression (score of ≥5).

### 2.3. Exposure Variable: Dietary Patterns 

NHANES uses 24 h dietary recall interviews to assess food and nutrient intake. Trained interviewers collected information on all the food and beverages consumed by participants in the previous 24 h, including portion sizes and preparation methods. The validated automated multiple pass method was used to ensure completeness and gather additional details on forgotten foods, preparation methods, ingredients, and condiments via probing [31]. Two 24 h recalls were obtained—the first one was collected in person, and the second recall was via a telephone interview 3–10 days following the first recall [32]. The data obtained from the 24 h recall method were used to estimate nutrient intakes of individuals. The obtained data were processed for nutrient analysis by using the United States Department of Agriculture’s (USDA) Food and Nutrient Database for Dietary Studies (FNDDS). The FNDDS is based on the USDA National Nutrient Database for Standard Reference, and it includes a variety of items such as branded and ethnic foods. It is updated every 2 years for the addition of any new food items and portion sizes as needed, using food labels or web sources, or by obtaining the information directly from manufacturers. 

Dietary patterns were constructed using factor analysis. Initially, all food items were collapsed into 28 food groups, similar to the grouping in the Food Patterns Equivalents Database (FPED) [33]. Food code from the USDA for an individual’s two-day dietary recalls of NHANES was compared to the USDA food code of FPED 2011–2012. As FPED 2011–2012 presents food and beverage components per 100 g, the grams of an individual’s food intake were divided by 100 g and multiplied by the corresponding number of FPED equivalents in FPED 2011–2012. The number of factors to retain was determined using three criteria: (1) examining the scree plot, which showed the eigenvalues for each factor in order from highest to lowest; (2) selecting only factors with eigenvalues greater than 1; and (3) interpretability of the identified patterns. Varimax rotation was used to help with the interpretation of the identified patterns, which minimizes the correlation between factors. The factor loadings were examined to identify the most important contributors in each dietary pattern. Each individual was assigned a factor score for each dietary pattern based on the factor loadings and actual food intake. The derived dietary patterns were named based on their resemblance to the prudent and Western patterns in existing publications and dietary guidelines.

### 2.4. Covariates

A priori selected covariates were included in the analysis. These were age, sex, ethnicity (Non-Hispanic White, Non-Hispanic Black, Mexican American, and Others), energy intake, physical activity (measured as Metabolic Equivalent of Task (METs) minutes per week and recoded into <600, 600–1200, and ≥1200 MET min/week), education (less than 11 grade, high school, some college, and higher than college), smoking status (never, former, current smoker), and alcohol. 

Alcohol drinking was assessed by self-reported alcohol consumption in the past 12 months and categorized as “yes” and “no”. Education level was assessed by the question “What is the highest grade or level of school completed or the highest degree received?” Physical activity was measured using data from the validated Global Physical Activity Questionnaire which includes questions on intensity, frequency and duration of daily physical activity. These were divided into 3 categories of MET minutes per week to rate their intensity as low, medium or high, as detailed above. Socioeconomic status (SES) was also assessed by dividing family income by the poverty threshold and categorizing it as <1.3, 1.3–3.5, and >3.5 Poverty Income Ratio. 

### 2.5. Statistical Analyses

Sample characteristics were presented as mean and standard deviation (SD) for continuous measures and *n* (%) for categorical measures by quartiles of dietary patterns. Chi-square test or ANOVA was used to test the difference for categorical or continuous variables by groups. Multivariable logistic regression was performed to examine the associations of CircS with dietary patterns, using the first quartile as the reference for each dietary pattern. To test the linear association for trend across the quartiles, the continuous dietary pattern score was used in the multivariable model. The multivariable models were adjusted for age, sex, ethnicity, educational level, physical activity, energy intake, smoking status, and alcohol consumption. To account for the complex survey design of NHANES data, appropriate sampling weights were used. Subgroup analyses to test for potential effect modification of CircS with diet patterns by various factors (age, sex, ethnicity, educational level, physical activity, energy intake, smoking status, and alcohol consumption) were conducted by adding product terms in the regression models. All the analyses were performed using STATA 17 (Stata Corporation, College Station, TX, USA). Statistical significance was considered when *p*-values were less than 0.05.

## 3. Results

### 3.1. Sample Characteristics

**Figure 2:**
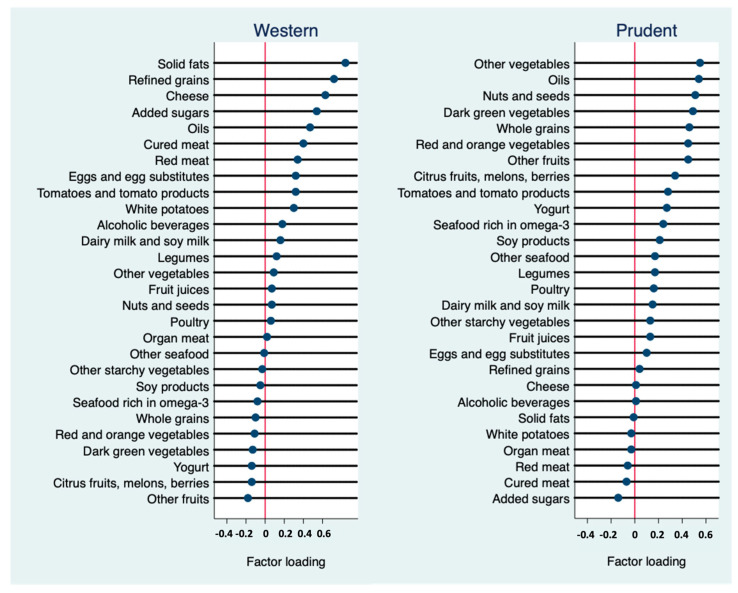
Factor loadings of the Western and prudent dietary patterns. Values are based on varimax rotation. Soy milk includes calcium–fortified soy milk.

A total number of 10,486 adults with a mean age of 50.3 years (SD 17.6) were included in the cross-sectional study. The unweighted prevalence of CircS was 41.3%. More than one third of the participants had short sleep (Supplementary Figure S1). Factor analysis identified two major dietary patterns in the study sample, and their factor loadings and corresponding scree plot are presented in Figure 2 and Supplementary Figure S2, respectively. The Western dietary pattern was characterized by a high intake of refined grains, solid fats, cheese, added sugars, cured meat, red meat, tomatoes and tomato products, eggs and egg substitutes, and white potatoes. Meanwhile, those on the prudent dietary pattern had a high intake of vegetables (red, orange, dark green, and others), oils, nuts and seeds, whole grains, fruits, yogurt, seafood, and soy products. The two dietary patterns explained 18.5% of the variance in food intake. 

Table 1 and Table 2 show the sample characteristics by quartiles of the Western and prudent dietary patterns. Those in the highest quartile of the Western pattern tended to be younger, men, current smokers, had higher energy intake, and were also more likely to report short sleep, whereas participants with the highest prudent pattern scores were more likely to be men, never smokers, had a higher level of education, and were slightly older and more active than those in the lowest quartile (Table 2). They also had a lower prevalence of MetS, depression symptoms, and short sleep.

### 3.2. Dietary Patterns and CircS and MetS

Across the quartiles of the Western dietary pattern, the unweighted prevalence of CircS was 45.2%, 41.6%, 40.6% and 37.8%, respectively. The corresponding figures were 42.8%, 44.4%, 42.3% and 35.7% across quartiles of the prudent dietary pattern, respectively.

The associations between dietary patterns and CircS are shown in Table 3. Higher Western pattern scores were positively associated with CircS, while the prudent pattern had an inverse association. In model 1, adjusting for age, sex, ethnicity, and energy intake, compared to those in the lowest quartile of the Western dietary pattern intake, participants in the highest quartile had significantly higher odds of CircS (OR = 2.39; 95%CI 1.85–3.09). The association was slightly attenuated after adjusting for lifestyle factors. When modeled as a continuous variable, the odds for CircS increased by 53% for each SD increment of the Western pattern score in the fully adjusted model (OR = 1.53; 95%CI 1.33–1.75; *p* < 0.001). In contrast, those in the highest quartile of the prudent dietary pattern had 29% lower odds of CircS in the fully adjusted model (OR = 0.71; 95%CI 0.58–0.86). 

The Western dietary pattern was positively associated with all the components of CircS except depressive symptoms and short sleep (Table 4). For each SD increment of Western dietary pattern score, the ORs were 1.68 (95%CI 1.48–1.91) for central obesity, 1.40 (95%CI 1.24–1.58) for elevated glucose, 1.27 (95%CI 1.13–1.42) for elevated triglyceride, 1.25 (95%CI 1.13–1.40) for low HDL, and 1.28 (95%CI 1.13–1.46) for elevated blood pressure. In contrast, the prudent dietary pattern was inversely associated with all the components of CircS (Table 4). Each SD increment of the prudent dietary pattern was associated with 7–17% decreased likelihood of having individual components of CircS.

Similar associations between dietary patterns and MetS were found (Supplementary Table S1). Each SD increment of Western pattern was associated with a 54% increase in MetS. In contrast, each SD increment of prudent pattern was associated with a 16% decrease in MetS.

### 3.3. Subgroup Analyses

Subgroup analyses were conducted to test whether the associations between the dietary patterns and CircS were modified by sociodemographic and lifestyle factors. There was a significant interaction between sex, income, education, and Western dietary pattern scores in relation to CircS. The association between Western dietary pattern and CircS was stronger among men and those with low income and high education (Table 5). Among those with higher education, higher Western dietary pattern scores were associated with an OR of 3.38 (95%CI 1.90–6.04). Similarly, an interaction between education, ethnicity and the prudent dietary pattern was found. The inverse association between the prudent dietary pattern and CircS was the strongest among those with a high education (Q4 had an OR of 0.46; 95%CI 0.31–0.69) and those of Non-Hispanic White descent (OR = 0.63; 95%CI 0.49–0.83 in Q4) (Table 6).

## 4. Discussion

In this large nationally representative sample, we found that the prevalence of CircS was high, affecting four out of ten adults in the US. Based on 2 days of 24 h diet recall, two dietary patterns were identified—Western and prudent dietary pattern. A positive relationship was observed between CircS and higher Western pattern scores, characterized by a high intake of refined grains, solid fats, added sugars, and red and cured meats. The association was stronger among men and those with low income and high education level. In contrast, the prudent pattern—high in vegetables, whole grains, oils, nuts, and seeds—was inversely associated with CircS. The inverse association was the strongest among Non-Hispanic White individuals and those with a high education level.

### 4.1. Comparison with Other Studies

To the best of our knowledge, this is the first study to assess the relationship between dietary patterns and CircS, defined by the addition of short sleep and depression symptoms to MetS. Previous studies have consistently reported similar associations of Western and prudent patterns with cardiometabolic risk traits. Three prior meta-analyses revealed that MetS was inversely associated with the healthy pattern and positively associated with the Western or unhealthy pattern [34,35,36]. 

Apart from cardiometabolic risk traits, similar associations of prudent dietary patterns with CircS components of short sleep [37] and depression [38] have also been reported. The Mediterranean Diet (MeDi) and a posteriori-derived prudent dietary pattern have been associated with improved sleep outcomes such as >6 h of sleep [39] and shorter sleep onset latency, respectively [40]. Similarly, a meta-analysis of observational research showed that higher adherence to the MeDi had the strongest evidence for reduced risk of developing depression [38]. Furthermore, an umbrella review of 28 meta-analyses of prospective studies showed that compared to low adherence, a higher adherence to a healthy diet was associated with reduced risk of depression. Notably, MeDi, vegetarian, and Western diets were not associated with depression risk; however, this was based on low quality of evidence [41].

### 4.2. Potential Mechanisms

In the present study, higher Western pattern scores were positively associated with all components of CircS, except for short sleep and depressive symptoms. Several potential mechanisms may explain the link between dietary patterns and CircS. Firstly, dietary patterns were strongly associated with lifestyle, including sleep patterns. Secondly, it is well known that the Western dietary pattern is positively associated with inflammation and obesity, which are risk factors for most of the components of CircS [4]. The common link in these metabolic alterations appears to be a disruption in circadian rhythms [6]. When the transcriptional pathways that regulate these rhythms are disrupted by light, feeding, or other signals, it can lead to the development of obesity, hypertension, insulin resistance, and dyslipidemia [6]. Irregular circadian rhythms have been implicated in obesity, diabetes, cardiovascular disease, and hypertension [42,43,44]. These chronic diseases are also commonly associated with sleep disturbances and depression [45,46,47]. The exact mechanisms of dietary patterns and their effect on circadian rhythms via clock gene expression are not yet fully understood. Studies on mice models suggest circadian disruption on a high-fat diet [48]. Prudent dietary pattern may exert protective effects against CircS through the inclusion of melatonin-rich foods, as melatonin is a sleep regulatory hormone that regulates rhythms of the sleep/wake cycle [49]. Additional factors not accounted for may explain some of the null associations observed, such as environmental factors, genetic polymorphisms, or the gut microbiota, which also has circadian rhythms [50]. 

Significant effects were observed in subgroup analyses of dietary patterns with CircS. Higher scores on the Western pattern were associated with CircS among men and those with a lower SES. It may be speculated that men are more likely to store excess visceral fat in the abdominal region, which is associated with higher MetS risk [51]. Additionally, they may be more likely to engage in behaviors contributing to weight gain, such as consuming more alcohol [52]. Low SES is also a risk factor for CVD due to social disparities and health inequities [53,54], which may explain the association with CircS in the current study. Interestingly, a modification effect of high education was observed in both dietary patterns. Higher education was associated positively with CircS on the Western pattern and had an inverse association on the prudent pattern. Existing literature shows that the relationship between education and diet quality seems to be linear [55]; however, it may be affected in the current study by the presence of substantially more men on the Western diet, who tend to have lower healthy eating index scores [56]. Furthermore, fast-paced lifestyles associated with higher education and high-stakes occupations may be conducive to consuming ready-to-eat, convenient foods on the go, with little nutritional value. An effect modification of ethnicity in relation to the prudent pattern and CircS was observed in this study. This could be attributable to the fact that Non-Hispanic White individuals tend to have a higher income bracket than other ethnicities in the study [57], and may be able to afford healthier lifestyles, hence the stronger inverse association with CircS on a prudent pattern.

### 4.3. Implications of Circadian Syndrome

Recently, CircS was proposed to highlight the underlying circadian rhythm disturbances linked to the cardiometabolic conditions that occur together in MetS and its associated comorbidities [6]. Shift work, social jet lag (defined as the discrepancy between sleep and wake times on weekdays vs. weekends), extended artificial light exposure, and eating at night have been associated with circadian disruption [6]. While circadian disruption has long been discussed in the literature, diagnosing it remains a challenge. CircS as a measure of circadian dysfunction is a recent concept that has been gaining attention, and its prevalence has been assessed in the Chinese and US populations. In a prospective study in China, CircS was found to be prevalent in 39% of the population at baseline [8]. A similar prevalence rate of approximately 41% was found in US adults [26], which is comparable to the current study. Emerging evidence on CircS indicates that it can have a profound impact on health, especially due to modern lifestyle behaviors—artificial light at night, increased use of electronic devices, and extended hours of food consumption—which are risk factors for circadian misalignment. These can disrupt the natural synchronization of the body’s internal clock with the external environment, leading to circadian rhythm disturbances [6]. The increasing prevalence of CircS and its implications highlight the need for interventions to mitigate the effects of circadian disruption on human health.

### 4.4. Strengths and Limitations 

The study has certain limitations that should be considered. Dietary intake was assessed using 24 h recall which may be associated with recall bias and might not adequately reflect usual intake. However, an average of two-day dietary recalls collected by a trained interviewer on separate occasions were used in the analysis. Another limitation of the study is the low variance explained by the two dietary patterns. The variance explained by the dietary patterns was determined by several factors including the number of food intake groups used in the analysis. In the study, we aggregated the food intake into 28 groups. However, the total variance explained by the two dietary patterns in our study was similar to other similar studies in the US [58]. Furthermore, there were no repeated measurements of CircS. Moreover, self-reported sleep duration was used rather than objective measures, which may impact the validity of the results. The threshold utilized for assessing symptoms of depression on the PHQ-9 was set at 5, rather than 10, which only accounts for mild symptoms of depression. The benefit of using PHQ-9 is its ease of use and time efficiency. Additionally, while NAFLD was originally proposed as part of the CircS, it was not included in the definition used in this study, similar to prior studies, for the sake of consistency and comparison. Although appropriate statistical models were used to control for confounding, residual confounding may still exist, and the cross-sectional design limits the ability to confer causality. This study has some notable strengths. The use of a large nationally representative sample enhances the ability to generalize the findings to the US population. Additionally, it is the first study to investigate the relationship of CircS with variables related to diet. As CircS appears to be a better predictor for CVD than MetS, our findings suggest that CircS may be the mediator for the association between dietary pattern and CVD. Further research is needed to test this hypothesis.

## 5. Conclusions

In conclusion, the prudent dietary pattern was inversely associated with CircS, while the Western pattern had a positive association with CircS in the current study. The study provides evidence for the use of CircS as a practical tool for measuring circadian disruption in clinical settings. However, further research including longitudinal studies is needed to validate these findings in different populations.

## Figures and Tables

**Table 1:** Sample characteristics by quartiles of the Western dietary pattern among participants attending NHANES 2005-2016 ^a^.

	Total	Q1	Q2	Q3	Q4	*p*-Value ^b^
	*n* = 10,486	*n* = 2622	*n* = 2621	*n* = 2622	*n* = 2621	
Energy intake (kcal/day)	2023.8 (781.8)	1299.9 (399.5)	1704.1 (360.3)	2120.1 (394.3)	2971.1 (683.7)	<0.001
Protein intake (g/day)	80.2 (34.0)	56.9 (24.6)	69.2 (23.2)	83.0 (25.3)	111.7 (34.4)	<0.001
Fat intake (g/day)	76.6 (36.3)	43.0 (16.7)	62.7 (16.8)	81.5 (19.0)	119.4 (34.0)	<0.001
Carbohydrate intake (g/day)	246.5 (100.5)	171.7 (63.2)	211.5 (62.9)	255.0 (69.2)	347.9 (102.3)	<0.001
Western pattern	0.0 (1.0)	−1.1 (0.3)	−0.4 (0.2)	0.2 (0.2)	1.4 (0.8)	<0.001
Prudent pattern	0.0 (1.0)	0.1 (1.1)	−0.1 (1.0)	−0.1 (0.9)	0.1 (1.0)	<0.001
Age (years)	50.3 (17.6)	55.5 (17.5)	52.6 (17.7)	49.1 (17.5)	44.0 (15.5)	<0.001
Sex						<0.001
Men	5147 (49.1%)	756 (28.8%)	1055 (40.3%)	1389 (53.0%)	1947 (74.3%)	
Women	5339 (50.9%)	1866 (71.2%)	1566 (59.7%)	1233 (47.0%)	674 (25.7%)	
Ethnicity						<0.001
Non-Hispanic White	4973 (47.4%)	1064 (40.6%)	1235 (47.1%)	1329 (50.7%)	1345 (51.3%)	
Non-Hispanic Black	2002 (19.1%)	536 (20.4%)	530 (20.2%)	461 (17.6%)	475 (18.1%)	
Mexican American	1587 (15.1%)	340 (13.0%)	361 (13.8%)	421 (16.1%)	465 (17.7%)	
Others	1924 (18.3%)	682 (26.0%)	495 (18.9%)	411 (15.7%)	336 (12.8%)	
Education						<0.001
<11 grade	2494 (23.8%)	690 (26.4%)	629 (24.0%)	589 (22.5%)	586 (22.4%)	
High school	2400 (22.9%)	571 (21.8%)	566 (21.6%)	599 (22.9%)	664 (25.3%)	
Some college	3043 (29.0%)	694 (26.5%)	763 (29.1%)	781 (29.8%)	805 (30.7%)	
Higher than college	2541 (24.3%)	662 (25.3%)	663 (25.3%)	651 (24.8%)	565 (21.6%)	
Smoking						<0.001
Never	5694 (54.3%)	1637 (62.5%)	1454 (55.5%)	1341 (51.2%)	1262 (48.1%)	
Former	2698 (25.7%)	632 (24.1%)	708 (27.0%)	710 (27.1%)	648 (24.7%)	
Current smoker	2090 (19.9%)	352 (13.4%)	457 (17.4%)	570 (21.7%)	711 (27.1%)	
Alcohol drinking (past 12 months)						<0.001
No	1938 (18.5%)	548 (20.9%)	525 (20.0%)	451 (17.2%)	414 (15.8%)	
Yes	7143 (68.1%)	1515 (57.8%)	1716 (65.5%)	1917 (73.1%)	1995 (76.1%)	
Missing	1405 (13.4%)	559 (21.3%)	380 (14.5%)	254 (9.7%)	212 (8.1%)	
BMI (kg/m^2^)	29.1 (6.7)	28.5 (6.5)	29.1 (6.7)	29.3 (6.8)	29.4 (6.9)	<0.001
Leisure time physical activity (METs minutes/week)						<0.001
<600	4153 (39.6%)	1172 (44.7%)	1121 (42.8%)	1015 (38.7%)	845 (32.3%)	
600–1200	1218 (11.6%)	335 (12.8%)	298 (11.4%)	322 (12.3%)	263 (10.0%)	
≥1200	5114 (48.8%)	1115 (42.5%)	1202 (45.9%)	1285 (49.0%)	1512 (57.7%)	
Ratio of family income to poverty						0.10
<1.30	2904 (29.9%)	758 (32.1%)	691 (28.6%)	701 (28.7%)	754 (30.4%)	
1.3–3.5	3717 (38.3%)	890 (37.6%)	941 (38.9%)	961 (39.4%)	925 (37.3%)	
>3.5	3084 (31.8%)	717 (30.3%)	785 (32.5%)	778 (31.9%)	804 (32.4%)	
Hypertension	3871 (37.0%)	1164 (44.5%)	1059 (40.4%)	913 (34.8%)	735 (28.1%)	<0.001
Central obesity	6056 (57.8%)	1588 (60.6%)	1572 (60.0%)	1529 (58.3%)	1367 (52.2%)	<0.001
Elevated glucose	5567 (53.1%)	1406 (53.6%)	1374 (52.4%)	1386 (52.9%)	1401 (53.5%)	0.81
Elevated triglycerides	4495 (42.9%)	1175 (44.8%)	1129 (43.1%)	1126 (42.9%)	1065 (40.6%)	0.024
Reduced HDL-C	4724 (45.1%)	1255 (47.9%)	1199 (45.7%)	1175 (44.8%)	1095 (41.8%)	<0.001
Elevated blood pressure	5137 (49.0%)	1449 (55.3%)	1348 (51.4%)	1242 (47.4%)	1098 (41.9%)	<0.001
Depression symptoms	2421 (23.1%)	626 (23.9%)	598 (22.8%)	592 (22.6%)	605 (23.1%)	0.70
Short sleep	3657 (34.9%)	889 (33.9%)	903 (34.5%)	870 (33.2%)	995 (38.0%)	0.001
Metabolic Syndrome	5124 (48.9%)	1383 (52.7%)	1318 (50.3%)	1261 (48.1%)	1162 (44.3%)	<0.001
Circadian Syndrome	4331 (41.3%)	1184 (45.2%)	1091 (41.6%)	1065 (40.6%)	991 (37.8%)	<0.001

^a^ Data were presented as mean (SD) for continuous measures, and *n* (%) for categorical measures. ^b^ *p* values were based on ANOVA for continuous measures and Chi-squared test for categorical measures. Abbreviations: BMI, Body Mass Index; METs, Metabolic Equivalents of Task; HDL-C, High-Density Lipoprotein Cholesterol.

**Table 2:** Sample characteristics by quartiles of the prudent dietary pattern among participants attending NHANES 2005–2016 ^a^.

	Total	Q1	Q2	Q3	Q4	*p*-Value ^b^
	*n* = 10,486	*n* = 2622	*n* = 2621	*n* = 2622	*n* = 2621	
Energy intake (kcal/day)	2023.8 (781.8)	1715.2 (691.1)	1911.2 (713.8)	2078.8 (723.8)	2389.9 (830.4)	<0.001
Protein intake (g/day)	80.2 (34.0)	64.0 (27.8)	74.4 (29.3)	83.4 (30.6)	98.8 (37.4)	<0.001
Fat intake (g/day)	76.6 (36.3)	63.1 (29.2)	71.6 (31.9)	78.8 (34.3)	93.1 (41.6)	<0.001
Carbohydrate intake (g/day)	246.5 (100.5)	212.9 (95.0)	234.0 (92.3)	252.7 (93.2)	286.6 (106.0)	<0.001
Western pattern	0.0 (1.0)	−0.0 (0.9)	0.0 (0.9)	0.0 (1.0)	0.0 (1.2)	0.009
Prudent pattern	0.0 (1.0)	−1.0 (0.2)	−0.5 (0.1)	0.1 (0.2)	1.3 (0.9)	<0.001
Age (years)	50.3 (17.6)	47.6 (17.9)	50.6 (18.2)	51.6 (17.4)	51.3 (16.5)	<0.001
Sex						<0.001
Men	5147 (49.1%)	1263 (48.2%)	1219 (46.5%)	1289 (49.2%)	1376 (52.5%)	
Women	5339 (50.9%)	1359 (51.8%)	1402 (53.5%)	1333 (50.8%)	1245 (47.5%)	
Ethnicity						<0.001
Non-Hispanic White	4973 (47.4%)	1247 (47.6%)	1166 (44.5%)	1249 (47.6%)	1311 (50.0%)	
Non-Hispanic Black	2002 (19.1%)	636 (24.3%)	535 (20.4%)	460 (17.5%)	371 (14.2%)	
Mexican American	1587 (15.1%)	349 (13.3%)	454 (17.3%)	432 (16.5%)	352 (13.4%)	
Others	1924 (18.3%)	390 (14.9%)	466 (17.8%)	481 (18.3%)	587 (22.4%)	
Education						<0.001
<11 grade	2494 (23.8%)	852 (32.6%)	713 (27.2%)	577 (22.0%)	352 (13.4%)	
High school	2400 (22.9%)	778 (29.7%)	652 (24.9%)	573 (21.9%)	397 (15.2%)	
Some college	3043 (29.0%)	713 (27.2%)	775 (29.6%)	782 (29.8%)	773 (29.5%)	
Higher than college	2541 (24.3%)	274 (10.5%)	481 (18.4%)	688 (26.3%)	1098 (41.9%)	
Smoking						<0.001
Never	5694 (54.3%)	1185 (45.2%)	1420 (54.2%)	1522 (58.0%)	1567 (59.8%)	
Former	2698 (25.7%)	534 (20.4%)	687 (26.2%)	686 (26.2%)	791 (30.2%)	
Current smoker	2090 (19.9%)	902 (34.4%)	512 (19.5%)	414 (15.8%)	262 (10.0%)	
Alcohol drinking (past 12 months)						<0.001
No	1938 (18.5%)	543 (20.7%)	513 (19.6%)	466 (17.8%)	416 (15.9%)	
Yes	7143 (68.1%)	1706 (65.1%)	1720 (65.6%)	1817 (69.3%)	1900 (72.5%)	
Missing	1405 (13.4%)	373 (14.2%)	388 (14.8%)	339 (12.9%)	305 (11.6%)	
BMI (kg/m^2^)	29.1 (6.7)	29.4 (7.1)	29.5 (6.9)	29.2 (6.5)	28.2 (6.3)	<0.001
Leisure time physical activity (METs minutes/week)						<0.001
<600	4153 (39.6%)	1194 (45.6%)	1137 (43.4%)	1034 (39.4%)	788 (30.1%)	
600–1200	1218 (11.6%)	269 (10.3%)	283 (10.8%)	320 (12.2%)	346 (13.2%)	
≥1200	5114 (48.8%)	1158 (44.2%)	1201 (45.8%)	1268 (48.4%)	1487 (56.7%)	
Ratio of family income to poverty						<0.001
<1.30	2904 (29.9%)	1015 (41.9%)	802 (32.9%)	611 (25.4%)	476 (19.6%)	
1.3–3.5	3717 (38.3%)	964 (39.8%)	1004 (41.1%)	942 (39.1%)	807 (33.2%)	
>3.5	3084 (31.8%)	445 (18.4%)	634 (26.0%)	855 (35.5%)	1150 (47.3%)	
Hypertension	3871 (37.0%)	916 (35.0%)	1040 (39.7%)	1018 (38.9%)	897 (34.2%)	<0.001
Central obesity	6056 (57.8%)	1552 (59.2%)	1609 (61.4%)	1545 (58.9%)	1350 (51.5%)	<0.001
Elevated glucose	5567 (53.1%)	1365 (52.1%)	1434 (54.7%)	1423 (54.3%)	1345 (51.3%)	0.034
Elevated triglycerides	4495 (42.9%)	1126 (42.9%)	1161 (44.3%)	1168 (44.5%)	1040 (39.7%)	0.001
Reduced HDL-C	4724 (45.1%)	1245 (47.5%)	1222 (46.6%)	1184 (45.2%)	1073 (40.9%)	<0.001
Elevated blood pressure	5137 (49.0%)	1266 (48.3%)	1387 (52.9%)	1298 (49.5%)	1186 (45.2%)	<0.001
Depression symptoms	2421 (23.1%)	769 (29.3%)	620 (23.7%)	567 (21.6%)	465 (17.7%)	<0.001
Short sleep	3657 (34.9%)	1036 (39.5%)	957 (36.5%)	869 (33.1%)	795 (30.3%)	<0.001
Metabolic Syndrome	5124 (48.9%)	1295 (49.4%)	1371 (52.3%)	1308 (49.9%)	1150 (43.9%)	<0.001
Circadian Syndrome	4331 (41.3%)	1122 (42.8%)	1164 (44.4%)	1108 (42.3%)	937 (35.7%)	<0.001

^a^ Data were presented as mean (SD) for continuous measures, and *n* (%) for categorical measures. ^b^ *p* values were based on ANOVA for continuous measures and Chi-squared test for categorical measures. Abbreviations: BMI, Body Mass Index; METs, Metabolic Equivalents of Task; HDL-C, High-Density Lipoprotein Cholesterol.

**Table 3:** Odds ratio (95%CI) for Circadian Syndrome by quartiles of dietary patterns and as a continuous variable among adults attending NHANES 2005–2016 (*n* = 10,486).

	Quartiles of Dietary Pattern					
	Q1	Q2	Q3	Q4	Intake as Continuous Variable (per 1 SD)	*p*-Value ^a^
Western pattern						
Unadjusted	1.00	0.82 (0.71–0.95)	0.85 (0.73–0.99)	0.84 (0.73–0.98)	0.96 (0.91–1.01)	0.105
Model 1	1.00	1.02 (0.87–1.20)	1.45 (1.18–1.77)	2.39 (1.85–3.09)	1.77 (1.54–2.03)	<0.001
Model 2	1.00	0.99 (0.84–1.15)	1.32 (1.08–1.60)	1.96 (1.53–2.53)	1.53 (1.33–1.75)	<0.001
Prudent pattern						
Unadjusted	1.00	1.05 (0.90–1.22)	0.92 (0.78–1.09)	0.74 (0.63–0.86)	0.85 (0.81–0.90)	<0.001
Model 1	1.00	0.85 (0.71–1.01)	0.66 (0.55–0.81)	0.49 (0.41–0.59)	0.74 (0.69–0.79)	<0.001
Model 2	1.00	0.97 (0.80–1.17)	0.83 (0.68–1.03)	0.71 (0.58–0.86)	0.84 (0.79–0.91)	<0.001

Values are odds ratios (95%CI) from logistic regression. Model 1 adjusted for age, sex, ethnicity, energy intake. Model 2 further adjusted for physical activity, education, smoking, and alcohol drinking. ^a^ *p* values were calculated using dietary intake as a continuous variable in multivariable models.

**Table 4:** Odds ratios (95%CI) for components of Circadian Syndrome by quartiles of dietary patterns among adults attending NHANES 2005–2016.

	Q1	Q2	Q3	Q4	Dietary Pattern as Continuous Variable (per 1 SD)	*p*-Value ^a^
	Western pattern					
Central obesity	1.00	1.25 (1.07–1.46)	1.71 (1.45–2.01)	2.22 (1.76–2.81)	1.68 (1.48–1.91)	<0.001
Elevated glucose	1.00	1.04 (0.88–1.22)	1.29 (1.09–1.53)	1.76 (1.35–2.30)	1.40 (1.24–1.58)	<0.001
Elevated triglyceride	1.00	1.03 (0.88–1.22)	1.18 (0.97–1.44)	1.45 (1.15–1.82)	1.27 (1.13–1.42)	<0.001
Low HDL	1.00	1.08 (0.92–1.26)	1.25 (1.05–1.49)	1.56 (1.27–1.92)	1.25 (1.13–1.40)	<0.001
Elevated blood pressure	1.00	0.91 (0.76–1.07)	1.13 (0.90–1.40)	1.28 (0.95–1.73)	1.28 (1.13–1.46)	<0.001
Depressive symptoms	1.00	0.88 (0.75–1.05)	0.95 (0.78–1.16)	1.04 (0.78–1.40)	1.08 (0.95–1.23)	0.254
Short sleep	1.00	0.94 (0.79–1.11)	0.89 (0.76–1.04)	1.13 (0.87–1.47)	1.09 (0.96–1.24)	0.173
	Prudent pattern					
Central obesity	1.00	0.98 (0.82–1.16)	0.89 (0.75–1.04)	0.70 (0.59–0.83)	0.83 (0.78–0.89)	<0.001
Elevated glucose	1.00	1.01 (0.87–1.17)	0.91 (0.78–1.06)	0.88 (0.74–1.06)	0.93 (0.87–0.99)	0.026
Elevated triglyceride	1.00	1.01 (0.85–1.19)	0.91 (0.77–1.08)	0.77 (0.64–0.92)	0.87 (0.81–0.92)	<0.001
Low HDL	1.00	0.90 (0.77–1.04)	0.80 (0.69–0.93)	0.78 (0.65–0.92)	0.89 (0.83–0.96)	0.002
Elevated blood pressure	1.00	1.18 (1.02–1.37)	0.87 (0.72–1.04)	0.75 (0.62–0.92)	0.83 (0.78–0.89)	<0.001
Depressive symptoms	1.00	0.75 (0.63–0.89)	0.76 (0.63–0.91)	0.68 (0.56–0.84)	0.87 (0.81–0.93)	<0.001
Short sleep	1.00	0.86 (0.75–0.99)	0.77 (0.65–0.91)	0.68 (0.58–0.80)	0.89 (0.83–0.95)	<0.001

Multivariable logistic regression models were adjusted for age, sex, ethnicity, energy intake, leisure time physical activity, education, smoking and alcohol drinking. ^a^ *p* values were calculated using dietary pattern as a continuous variable in multivariable models.

**Table 5:** Subgroup analyses of the association between quartiles of Western dietary pattern and Circadian Syndrome.

	Western Pattern					
	Q1	Q2	Q3	Q4	*p* for Trend	*p* for Interaction
Ethnicity						0.247
Non–Hispanic White	1.00	0.93 (0.75–1.16)	1.29 (0.99–1.67)	2.02 (1.43–2.87)	<0.001	
Non–Hispanic Black	1.00	1.17 (0.87–1.57)	1.17 (0.76–1.78)	1.86 (1.16–2.98)	0.104	
Mexican American	1.00	1.22 (0.80–1.86)	1.18 (0.72–1.93)	1.71 (0.91–3.21)	0.037	
Others	1.00	1.01 (0.68–1.48)	1.72 (1.10–2.71)	1.40 (0.72–2.70)	0.045	
Sex						0.021
Men	1.00	1.00 (0.75–1.33)	1.39 (1.04–1.87)	2.05 (1.48–2.85)	<0.001	
Women	1.00	1.00 (0.80–1.25)	1.26 (0.91–1.76)	1.80 (1.21–2.69)	<0.001	
Age group						0.063
20–39	1.00	1.32 (0.92–1.90)	1.57 (1.07–2.32)	2.50 (1.60–3.93)	0.004	
40–59	1.00	0.92 (0.69–1.23)	1.22 (0.89–1.66)	1.68 (1.13–2.48)	<0.001	
60+	1.00	1.01 (0.81–1.26)	1.53 (1.12–2.07)	2.45 (1.40–4.27)	<0.001	
Ratio of family income to poverty						<0.001
<1.30	1.00	1.10 (0.83–1.46)	1.21 (0.89–1.66)	1.94 (1.27–2.96)	0.011	
1.3–3.5	1.00	1.04 (0.77–1.41)	1.16 (0.86–1.56)	1.83 (1.24–2.71)	0.001	
>3.5	1.00	0.95 (0.71–1.28)	1.41 (0.97–2.05)	1.87 (1.13–3.07)	<0.001	
Education						<0.001
<11 grade	1.00	0.82 (0.57–1.19)	1.23 (0.83–1.82)	1.37 (0.81–2.31)	0.044	
High school	1.00	0.72 (0.52–1.01)	0.98 (0.65–1.48)	1.52 (0.92–2.51)	0.106	
Some college	1.00	0.89 (0.64–1.22)	1.20 (0.84–1.73)	1.62 (1.06–2.48)	<0.001	
Higher than college	1.00	1.65 (1.16–2.35)	1.93 (1.20–3.10)	3.38 (1.90–6.04)	<0.001	
Leisure time physical activity (METs minutes/week)						0.969
<600	1.00	0.95 (0.75–1.22)	1.19 (0.88–1.62)	1.76 (1.16–2.69)	<0.001	
600–1200	1.00	1.10 (0.68–1.79)	2.00 (1.18–3.40)	3.02 (1.40–6.49)	0.004	
≥1200	1.00	0.98 (0.74–1.31)	1.29 (0.97–1.73)	1.87 (1.27–2.75)	<0.001	
Smoking						0.116
Never	1.00	1.08 (0.86–1.36)	1.50 (1.12–1.99)	2.30 (1.63–3.24)	<0.001	
Former	1.00	0.90 (0.65–1.26)	1.20 (0.83–1.73)	1.60 (1.05–2.44)	<0.001	
Current smoker	1.00	0.82 (0.54–1.26)	1.00 (0.65–1.53)	1.69 (0.98–2.92)	0.362	

Multivariable logistic regression models were adjusted for age, sex, ethnicity, energy intake, leisure time physical activity, education, smoking and alcohol drinking. Stratification variables were not adjusted in the corresponding models.

**Table 6:** Subgroup analyses of the association between quartiles of prudent dietary pattern and Circadian Syndrome.

	Prudent Pattern					
	Q1	Q2	Q3	Q4	*p* for Trend	*p* for Interaction
Ethnicity						0.003
Non–Hispanic White	1.00	0.93 (0.74–1.17)	0.77 (0.59–1.00)	0.63 (0.49–0.83)	<0.001	
Non–Hispanic Black	1.00	1.15 (0.84–1.57)	1.34 (0.98–1.83)	1.14 (0.81–1.61)	0.499	
Mexican American	1.00	0.99 (0.68–1.43)	0.82 (0.54–1.25)	0.85 (0.53–1.37)	0.430	
Others	1.00	0.95 (0.58–1.57)	0.90 (0.55–1.48)	0.84 (0.49–1.42)	0.170	
Sex						0.078
Men	1.00	0.93 (0.72–1.20)	0.91 (0.71–1.18)	0.66 (0.52–0.85)	<0.001	
Women	1.00	1.06 (0.83–1.34)	0.79 (0.59–1.06)	0.81 (0.60–1.08)	0.039	
Age group						0.526
20–39	1.00	0.85 (0.59–1.23)	0.85 (0.61–1.19)	0.66 (0.43–1.02)	0.018	
40–59	1.00	1.10 (0.84–1.45)	0.91 (0.65–1.26)	0.73 (0.55–0.96)	0.004	
60+	1.00	0.85 (0.64–1.14)	0.69 (0.51–0.93)	0.64 (0.48–0.86)	<0.001	
Ratio of family income to poverty						0.196
<1.30	1.00	0.83 (0.64–1.08)	0.88 (0.63–1.23)	0.65 (0.45–0.94)	0.055	
1.3–3.5	1.00	0.84 (0.65–1.09)	0.84 (0.63–1.12)	0.83 (0.59–1.17)	0.080	
>3.5	1.00	1.51 (1.04–2.19)	1.04 (0.73–1.50)	0.83 (0.59–1.18)	<0.001	
Education						0.002
<11 grade	1.00	0.81 (0.62–1.05)	0.83 (0.63–1.11)	0.86 (0.58–1.28)	0.370	
High school	1.00	0.94 (0.69–1.28)	0.94 (0.66–1.33)	0.97 (0.66–1.43)	0.917	
Some college	1.00	0.97 (0.68–1.38)	0.96 (0.70–1.32)	0.80 (0.61–1.07)	0.010	
Higher than college	1.00	1.04 (0.70–1.55)	0.58 (0.39–0.88)	0.46 (0.31–0.69)	<0.001	
Leisure time physical activity (METs minutes/week)						0.126
<600	1.00	0.96 (0.72–1.27)	0.81 (0.63–1.05)	0.79 (0.59–1.07)	0.114	
600–1200	1.00	0.80 (0.50–1.26)	0.80 (0.45–1.42)	0.79 (0.46–1.38)	0.482	
≥1200	1.00	0.98 (0.75–1.28)	0.83 (0.63–1.10)	0.64 (0.48–0.86)	<0.001	
Smoking						0.178
Never	1.00	1.12 (0.89–1.42)	0.88 (0.70–1.11)	0.69 (0.53–0.90)	<0.001	
Former	1.00	0.69 (0.49–0.98)	0.59 (0.43–0.81)	0.58 (0.41–0.82)	0.004	
Current smoker	1.00	1.01 (0.72–1.43)	1.13 (0.75–1.71)	1.02 (0.64–1.62)	0.818	

Multivariable logistic regression models were adjusted for age, sex, ethnicity, energy intake, leisure time physical activity, education, smoking and alcohol drinking. Stratification variables were not adjusted in the corresponding models.

## Data Availability

The data used in the study are publicly available from the NHANES website.

## Supplementary Materials

The following supporting information can be downloaded at: https://www.mdpi.com/article/10.3390/nu15153396/s1, Figure S1: Distribution of sleep duration; Figure S2: Scree plot of factor analysis; Table S1: Odds ratio (95%CI) for Metabolic Syndrome by quartiles of dietary patterns and as a continuous variable among adults attending NHANES 2005-2016 (*n* = 10,486); Table S2: Odds ratios (95%CI) for components of Metabolic Syndrome by quartiles of dietary patterns among adults attending NHANES 2005-2016.
