# Supplementary material for: Dietary Patterns and Circadian Syndrome among Adults Attending NHANES 2005–2016
Source: Nutrients. 2023 Jul 31;15(15):3396. doi: 10.3390/nu15153396 (PMC10421411; doi:10.3390/nu15153396)
Supplement: Supplementary file 1 [file nutrients-15-03396-s001.zip › nutrients-2493133-supplementary.pdf]

Supplement Figure 1: Distribution of sleep duration

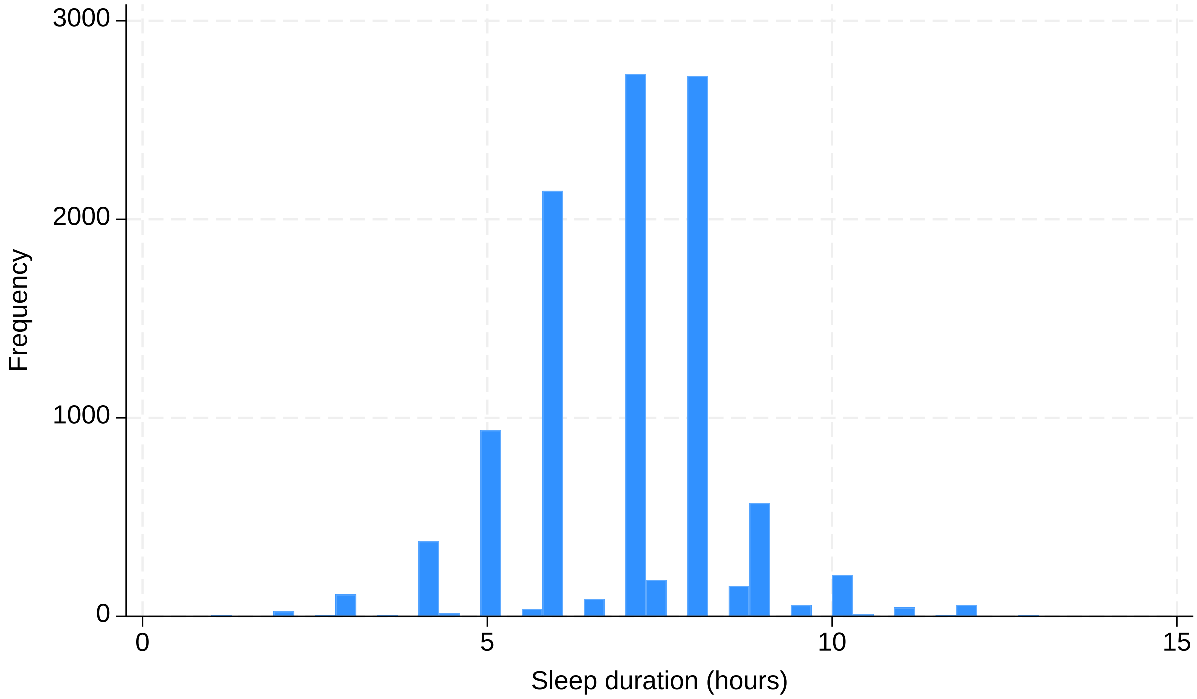

Supplement Figure 2: Scree plot of factor analysis.

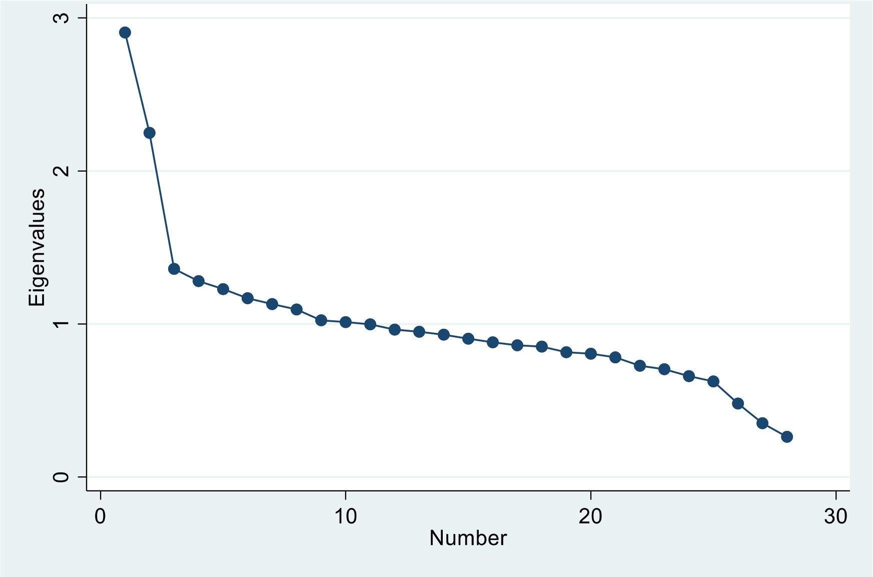

Supplement Table 1. Odds ratio (95%CI) for Metabolic Syndrome by quartiles of dietary patterns and as a continuous variable among adults attending NHANES 2005-2016 (n=10,486).

	Quartiles of dietary pattern					
	**Q1**	**Q2**	**Q3**	**Q4**	**Intake as continuous variable (per 1 SD)**	**p-value[a]**
Western pattern						
Unadjusted	1.00	0.88 (0.76-1.02)	0.86 (0.74-0.99)	0.82 (0.71-0.95)	0.94 (0.89-0.98)	0.008
Model 1	1.00	1.12 (0.95-1.31)	1.46 (1.22-1.76)	2.39 (1.85-3.07)	1.73 (1.52-1.97)	<0.001
Model 2	1.00	1.09 (0.93-1.28)	1.36 (1.13-1.64)	2.04 (1.58-2.62)	1.54 (1.34-1.76)	<0.001
Prudent pattern						
Unadjusted	1.00	1.13 (0.99-1.29)	0.98 (0.86-1.12)	0.81 (0.69-0.95)	0.88 (0.84-0.93)	<0.001
Model 1	1.00	0.93 (0.79-1.10)	0.71 (0.61-0.82)	0.54 (0.45-0.66)	0.77 (0.72-0.83)	<0.001
Model 2	1.00	1.02 (0.85-1.21)	0.83 (0.71-0.98)	0.71 (0.58-0.88)	0.85 (0.79-0.92)	<0.001

Values are odds ratios (95%CI) from logistic regression. Model 1 adjusted for age, sex, race, energy intake. Model 2 further adjusted for physical activity, education, smoking, and alcohol drinking.

[a]p values correspond to dietary intake as a continuous variable.

Supplement Table 2. Odds ratios (95%CI) for components of Metabolic Syndrome by quartiles of dietary patterns among adults attending NHANES 2005-2016.

	**Q1**	**Q2**	**Q3**	**Q4**	**Dietary pattern as continuous variable (per 1 SD)**	**p-value**
			Western pattern			
Central obesity	1.00	1.24 (1.06-1.45)	1.74 (1.46-2.08)	2.45 (1.93-3.11)	1.69 (1.48-1.93)	<0.001
Elevated blood pressure	1.00	0.96 (0.82-1.13)	1.21 (0.98-1.50)	1.53 (1.15-2.03)	1.31 (1.15-1.49)	<0.001
Elevated glucose	1.00	1.05 (0.90-1.24)	1.35 (1.14-1.60)	1.94 (1.50-2.51)	1.38 (1.22-1.56)	<0.001
Elevated triglyceride	1.00	1.06 (0.90-1.25)	1.29 (1.07-1.55)	1.69 (1.36-2.11)	1.28 (1.15-1.42)	<0.001
Low HDL	1.00	1.09 (0.94-1.26)	1.35 (1.15-1.60)	1.84 (1.50-2.25)	1.28 (1.15-1.42)	<0.001
			Prudent pattern			
Central obesity	1.00	0.99 (0.85-1.16)	0.85 (0.73-0.98)	0.63 (0.54-0.74)	0.83 (0.77-0.89)	<0.001
Elevated blood pressure	1.00	1.11 (0.97-1.28)	0.77 (0.64-0.92)	0.60 (0.50-0.72)	0.83 (0.78-0.89)	<0.001
Elevated glucose	1.00	0.96 (0.83-1.10)	0.82 (0.71-0.95)	0.75 (0.63-0.89)	0.94 (0.88-1.00)	0.055
Elevated triglyceride	1.00	0.95 (0.81-1.10)	0.79 (0.68-0.92)	0.62 (0.53-0.73)	0.86 (0.80-0.91)	<0.001
Low HDL	1.00	0.83 (0.72-0.97)	0.70 (0.60-0.81)	0.61 (0.52-0.72)	0.88 (0.82-0.95)	<0.001

Multivariable logistic regression models were adjusted for age, sex, ethnicity, energy intake, leisure time physical activity, education, smoking and alcohol drinking.
